# Behavior of Nutritional Supplements Use in Association With Inflammatory Skin Diseases in Chinese College Students

**DOI:** 10.3389/fnut.2021.615462

**Published:** 2021-03-17

**Authors:** Yan Yuan, Juan Su, Ji Li, Juan Tao, Xiaojing Kang, Bin Wu, Shijun Shan, Xiaohui Wang, Xiang Chen, Minxue Shen, Liyuan Jiang

**Affiliations:** ^1^The Department of Dermatology, Xiangya Hospital, Central South University, Changsha, China; ^2^Hunan Key Laboratory of Skin Cancer and Psoriasis, Xiangya Hospital, Central South University, Changsha, China; ^3^Hunan Engineering Research Center of Skin Health and Disease, Xiangya Hospital, Central South University, Changsha, China; ^4^Department of Dermatology, Tongji Medical College, Union Hospital, Huazhong University of Science and Technology, Wuhan, China; ^5^Department of Dermatology, People's Hospital of Xinjiang Uygur Autonomous Region, Urumchi, China; ^6^Department of Dermatology, The Affiliated People's Hospital of Inner Mongolia Medical University, Hohhot, China; ^7^Department of Dermatology, Xiang'an Hospital, Xiamen University, Xiamen, China; ^8^Department of Dermatology, Zhongshan Hospital, Xiamen University, Xiamen, China; ^9^Department of Social Medicine and Health Management, Xiangya School of Public Health, Central South University, Changsha, China; ^10^Mobile Joint Laboratory, Xiangya Hospital, Central South University, Changsha, China

**Keywords:** nutritional supplements use, inflammatory skin disease, college students, behavior, NS use

## Abstract

**Objectives:** It is understudied how frequently adolescents use nutritional supplements (NS) and whether the corresponding behavior is associated with skin diseases that may cause unpleasant symptoms and disfigurement. The current study aimed to investigate the prevalence of NS use in Chinese college students and its association with inflammatory skin diseases.

**Methods:** This was a university-based epidemiologic investigation that included 20,138 students who underwent dermatological examinations. A questionnaire survey was conducted to inquire about the use of NS along with related information. Skin diseases were diagnosed by dermatologists during the health examination. Logistic regression models were used for analysis. Adjusted odds ratios (aORs) were presented as the effect size.

**Results:** Survey responses from a total of 20,138 participants were analyzed. Specifically, 18.3% of the participants reported the use of NS in the past year. The use of vitamin C was most frequently reported, accounting for a proportion of 12.9%, followed by vitamin B and mineral supplements. The use of NS was found to be associated with female sex, Han ethnicity, higher annual household income, and a series of healthy lifestyles such as more physical activity, less second-hand smoke exposure, less alcohol consumption, and higher intake of milk and yogurt (*p* < 0.001). Participants with chronic urticaria (aOR = 1.3; 95% CI, 1.0–1.7), atopic dermatitis (aOR = 1.4; 95% CI, 1.2–1.6), or acne (aOR = 1.17; 95% CI, 1.04–1.31) were more likely to use NS, especially herbs (aOR = 2.7; 95% CI, 1.2–3.7), followed by vitamin B (aOR = 1.6; 95% CI, 1.2–2.0) and mineral supplements (aOR = 1.4; 95% CI, 1.0–2.0).

**Conclusion:** College students with inflammatory skin diseases are more likely to use NS.

## Introduction

Nutritional supplement (NS) refers to a kind of healthcare product extracted from one or several chemical syntheses, natural plants, or animal ingredients. It usually contains some of the following nutrients: vitamins, minerals, fiber, proteins, etc. (1). NS use is generally popular in the USA, with prevalence rates varying from 21 to 55% reported by several different studies (2). Our previous study revealed that only 0.71% of the participants used NS within 1 month before the survey (3).

Evidence suggests that demographic characteristics, lifestyles, and health characteristics were significantly associated with the overall prevalence of NS use. It is well-known that individuals affected by certain chronic diseases often use complementary and alternative medicines [CAMs, which can be defined as “forms of treatment that are used in addition to (complementary) or instead of (alternative) standard treatments”] as a way to relieve the progression of their diseases, manage comorbidities, and intensify perceived control over their health condition. As a matter of fact, NS is one of the frequently used CAMs.

Atopic dermatitis (AD) is a chronic inflammatory skin condition involving complex interactions between immunologic, hereditary, and environmental influences (4), affecting up to 20% of children and 3% of adults worldwide over the last 30 years (5). Urticaria, manifesting as recurrent itchy wheals and/or angioedema, affects 0.5–1% of the general population (6), and 0.1–0.3% of children (7). Rosacea is a chronic inflammatory disease with multiple skin manifestations, including transient or persistent erythema, papules, pustules, recurrent flushing, telangiectasia, and probably phymatous changes (8). The prevalence of rosacea is estimated to vary from 1 to 22% across different reports (9–11). Acne vulgaris is a chronic inflammatory disease that affects 79–95% of the adolescent population in Western countries (12).

At present, there is a paucity of up-to-date studies reporting the prevalence of NS use among China's college students and elaborating whether NS use is more common among individuals with inflammatory skin diseases.

## Methods

### Study Design

This was a cross-sectional study performed at five comprehensive universities in China. All newly enrolled students who consented to participate in this study underwent a health examination and responded to an online questionnaire between September and October 2018. More details of the sample and sampling process can be found in our previous studies (13–16).

### Questionnaire

A web-based questionnaire survey took place on a single day, organized by the Department of Student Affairs of the university. The participant freshmen completed the questionnaire in separate computer rooms where privacy was guaranteed. During the survey, three investigators were assigned to each room to provide technical support. The questionnaire consisted of 84 questions, including demographic information (ethnicity, original region, household annual income in yuan), disease history (cardiovascular/metabolic diseases, autoimmune diseases, infectious diseases, mental disorders, etc.), and behavior characteristics (cigarette smoking, passive smoking, alcohol drinking, soft drink intake, water intake, exercise, etc.). Within the questionnaire, one self-reported NS use items were asked: “Within the last year, which supplements were frequently used?” six supplements were listed as options for this item, including vitamin B, vitamin C, vitamin E, mineral, protein, and herbal. Participants were instructed to write in supplements they used.

### Clinical Evaluation and Diagnosis

The diagnosis of skin diseases and inquiries regarding disease history were performed by certified dermatologists during the health examination. For recurrent skin diseases, only those with current symptoms and lesions were considered cases of point prevalence. Height and weight were measured using standardized methods, which were used then to calculate the body mass index.

### Statistical Analysis

Continuous data were presented as mean ± standard deviation, and the between-group differences were tested using analysis of variance. Categorical data were presented as an absolute number in form of percentage (%), and the between-group differences were tested using the Chi-square test. Logistic models were used to estimate the effects of inflammatory skin diseases on the behavior of NS use. Odds ratios (ORs) and 95% confidence intervals (CIs) were estimated from the model. Adjusted odds ratios (aORs) were further estimated by adjusting for covariates [adjustment included level-1 confounders (age, gender, region, ethnicity, and annual household income) and level-2 confounder (daily alcohol drinking, intake of milk and yogurt, passive smoking, sport, sedentary activities)]. Statistical analysis was performed using SPSS 25.0 (IBM SPSS, Chicago, IL), and *P* < 0.05 was considered statistically significant.

## Results

A total of 20,138 students underwent the dermatological examination and completed a specially designed questionnaire after giving consent to this cross-sectional study. Of the participants, 3,703 (18.3%) reported the use of NS in the past year (see Supplementary Table 1 for the use of each type of NS). The use of vitamin C was most frequently reported, accounting for a proportion of 12.9%, followed by vitamin B and mineral supplements.

The characteristics of the participants stratified by the prevalence of NS use are shown in Table 1. The use of NS was found to be associated with female sex, Han ethnicity, higher annual household income, and a series of healthy lifestyles including more physical activity, less second-hand smoke exposure, less alcohol consumption, and higher intake of milk and yogurt (*P* < 0.001).

**Table 1:** Participant characteristics by the use of nutritional supplements.

**Variables**	**Total**	**Nutritional supplements**		
	**(*N* = 20,138)**	**No (*n* = 16,435)**	**Yes (*n* = 3,703)**	***P-*value**
Geographic region*, %				<0.001
North	17.9	18.0	17.3	
Northeast	3.1	2.8	4.6	
East	22.4	22.2	23.5	
Central	21.1	21.4	19.6	
South	6.7	6.8	6.3	
Southwest	8.9	9.3	7.5	
Northwest	19.8	19.5	21.2	
Age (year), mean ± SD	18.3 ± 0.8	18.3 ± 0.8	18.3 ± 0.7	0.251
BMI (kg/m^2^), %				0.230
Underweight (<18.5)	20.0	19.8	21.2	
Normal (18.5–23.9)	61.5	61.6	60.6	
Overweight (24.0–27.9)	13.3	13.3	13.3	
Obese (≥28.0)	5.2	5.2	4.9	
Sex, %				<0.001
Male	51.1	52.5	45.0	
Female	48.9	47.5	55.0	
Ethnicity, %				0.030
Han	80.6	80.3	81.9	
Other	19.4	19.7	18.1	
Annual household income (yuan), %				<0.001
<10,000	10.8	11.5	7.2	
10,000–29,999	21.7	23.0	16.2	
30,000–49,999	17.2	17.7	15.3	
50,000–99,999	21.9	21.8	22.8	
100,000–199,999	20.3	18.9	25.8	
≥200,000	8.1	7.1	12.7	
Frequency of physical activity (h/wk), %				0.039
0–2	42.1	42.5	40.5	
2–7	21.1	21.1	21.0	
≥7	36.8	36.4	38.5	
Sedentary activities (h/d), %				0.211
Hardly	7.9	8.0	7.6	
<7	45.3	45.5	44.2	
≥7	46.8	46.5	48.1	
Second hand smoke exposure, %				<0.001
Hardly	78.9	79.7	75.3	
Frequently	21.1	20.3	24.7	
Alcohol, %				<0.001
Hardly	95.5	95.9	93.9	
Frequently	4.5	4.1	6.1	
Milk (day/wk), %				<0.001
<1	31.1	32.8	23.8	
1–3	31.3	31.5	30.1	
>3	37.6	35.7	46.1	
Yogurt (day/wk), %				<0.001
<1	27.5	29.2	19.9	
1–3	44.0	44.3	42.9	
>3	28.5	26.5	37.2	

* *North: Beijing, Tianjin, Hebei, Shanxi, Inner Mongolia; Northeast: Liaoning, Jilin, Heilongjiang; East: Shanghai, Jiangsu, Zhejiang, Anhui, Fujian, Jiangxi, Shandong, Taiwan; Central: Henan, Hubei, Hunan; South: Guangdong, Guangxi, Hainan, Hong Kong, Macao; Southwest: Chongqing, Sichuan, Guizhou, Yunnan, Tibet; Northwest: Shaanxi, Gansu, Qinghai, Ningxia, Xinjiang*.

The association between skin diseases and NS use was examined. As shown in Table 2, participants with inflammatory skin diseases, such as CU (*P*_trend_ = 0.002) and AD (*P*_trend_ = 0.001), were associated with a higher prevalence of NS use, especially vitamin C, followed by vitamin B and mineral supplements. By contrast, the association between acne or rosacea and the prevalence of NS use was statistically insignificant (*P*_trend_ > 0.05).

**Table 2:** Use of nutritional supplements in participants with and without inflammatory skin diseases.

	**NS (%)**	**Vitamin B (%)**	**Vitamin C (%)**	**Vitamin E (%)**	**Mineral (%)**	**Protein (%)**	**Herbal (%)**
Atopic dermatitis							
Case	24.4	8.4	16.6	3.9	5.0	3.1	1.7
Control	18.1	5.0	12.7	3.0	3.4	2.6	0.7
*P*-value	<0.001	<0.001	0.001	0.188	0.017	0.476	0.002
Chronic urticarial							
Case	23.6	7.1	16.5	3.4	3.4	3.1	1.3
Control	18.3	5.1	12.8	3.0	3.1	2.6	0.7
*P*-value	0.008	0.112	0.036	0.754	0.866	0.63	0.325
Acne							
Case	19.9	6.2	14.2	3.7	4.5	2.6	1.1
Control	18.2	5.0	12.7	2.9	3.3	2.7	0.7
*P*-value	0.058	0.021	0.059	0.064	0.005	0.808	0.117
Rosacea							
Case	19.2	4.8	12.5	3.3	4.0	2.9	1.2
Control	18.4	5.2	12.9	3.0	3.4	2.6	0.7
*P*-value	0.586	0.694	0.764	0.698	0.413	0.638	0.305

*NS, nutritional supplements*.

The association of NS use with inflammatory skin diseases was then further adjusted for covariates. As shown in Table 3, the crude and adjusted estimates demonstrated a good consistency. After adjustments for demographic and behavioral factors, the results remained consistent: participants with chronic urticaria (aOR = 1.3; 95% CI, 1.0–1.7), atopic dermatitis (aOR = 1.4; 95% CI, 1.2–1.6), or acne (aOR = 1.17; 95% CI, 1.04–1.31) were more likely to use NS, especially herbs (aOR = 2.7; 95% CI, 1.2–3.7), followed by vitamin B (aOR = 1.6; 95% CI, 1.2–2.0) and mineral supplements (aOR = 1.4; 95% CI, 1.0–2.0).

**Table 3:** Adjusted odds ratios for the association of inflammatory skin diseases and use of nutritional supplements*.

**Disease**	**NS**	**Vitamin B**	**Vitamin C**	**Vitamin E**	**Mineral**	**Protein**	**Herbal**
Atopic dermatitis	1.4 (1.2, 1.6)^$^	1.6 (1.2, 2.0)^$^	1.3 (1.0, 1.6)^#^	1.1 (0.8, 1.7)	1.4 (1.0, 2.0)^#^	1.2 (0.8, 1.9)	2.1 (1.2, 3.7)^#^
Chronic urticaria	1.3 (1.0, 1.7)^#^	1.2 (0.9, 1.9)	1.3 (1.0, 1.7)	1.0 (0.6, 1.8)	0.9 (0.5, 1.5)	1.3 (0.7, 2.3)	1.5 (0.6, 3.8)
Acne	1.2 (1.0, 1.3)^$^	1.2 (1.0, 1.5)^#^	1.2 (1.0, 1.4)^$^	1.3 (1.0, 1.6)^#^	1.3 (1.1, 1.7)^#^	1.0 (0.8, 1.4)	1.6 (1.0, 2.5)
Rosacea	1.0 (0.8, 1.1)	0.8 (0.6, 1.2)	0.9 (0.7, 1.1)	1.0 (0.7, 1.5)	1.2 (0.8, 1.7)	1.3 (0.8, 1.9)	1.4 (0.7, 2.8)

*NS, nutritional supplements*.

* *Adjusted for demographic (age, gender, region, ethnicity and annual household income) and behavioral factors (daily alcohol drinking, intake of milk and yogurt, passive smoking, sport, sedentary activities)*.

# *P < 0.05*.

$ *P < 0.01*.

## Discussion

This was the first study that provided detailed information on NS use among Chinese college students, and that linked inflammatory skin diseases, especially atopic conditions, acne, and urticaria, with NS use in China.

The overall prevalence of NS use was 18.4% among college students. This finding was higher than other surveys in China (2) but lower than those in other countries (17–22). A lower prevalence was also reported among preschool children in China (2) compared with the USA (23, 24). NS use was more popular in developed countries, which might be attributed to the emphasis of using NS to ensure nutrient adequacy by the Dietary Guidelines (25). However, NS is not recommended for the general population, while acquiring adequate and comprehensive natural nutrients from daily diet is prioritized by the guidelines (26). The most popular NS consumed by college students in our study was vitamin C, followed by vitamin B and mineral supplements, and similar trends were also reported by other studies (27, 28).

Demographic characteristics were found to be associated with the overall prevalence of NS use. Specifically, NS use varies by sex, geography, ethnicity, and annual household income, which serves as a surrogate for one's socioeconomic status; for example, participants with a higher level of annual household income and purchasing power have better accessibility to NS and stronger healthcare consciousness, and as a result, more of them use NS (29).

The current study suggested that NS use was associated with health-related characteristics and might serve as a marker for a range of other health-related behaviors, evidenced by lower prevalence rates of passive smoking and alcohol drinking, higher frequency of sports activity, and higher intake of milk and yogurt, which was similar to the earlier-reported tendency for smoking, BMI, and physical activity; NS users have been characterized as having a positive attitude toward their health. In addition, this study showed that participants suffering from inflammatory skin diseases were more likely to use NS. This result was consistent with the findings of previous research (30, 31). Apart from inflammatory skin diseases, NS use had also been associated with a variety of other diseases (32), such as cancer and cardiac disease. This may be attributable to the fact that people with health problems have stronger health awareness and are more likely to change their lifestyles to improve health conditions (30, 31). Among the various skin diseases, participants with AD reported the highest proportion of NS use. This may be related to the public awareness that certain nutrients can reduce oxidative damage to cells and tissues (33–35) by neutralizing reactive oxygen species (36). In contrast, no association was found between rosacea and NS use. A possible reason is that rosacea is not a well-known skin problem by the public, and many patients would never visit a dermatologist for rosacea owing to subclinical or atypical symptoms or even misdiagnosis (37).

This study has some limitations. First, data was not collected on the amount of NS use. However, with our preliminary result, further study with quantitative measurements of NS on skin diseases are expecting. Second, since our sample was confined to college students with similar educational backgrounds, there might be a selection bias in representing general adolescents across China. Third, our hypothesis that patients with inflammatory diseases are more likely to use NS could not be confirmed owing to the cross-sectional study design.

The strengths of this study are more worth mentioning. First, this was a large-sample and multicenter study. Second, it was the first population-based study that linked inflammatory skin diseases with NS use in China. A comprehensive understanding of the characteristics of NS users can help develop effective public health interventions to reduce unnecessary consumption of NS. Third, this study had a high response rate and high completeness of questionnaires, and the ascertainment of skin diseases was performed by dermatologists, resulting in satisfying quality of evidence.

In summary, by examining the prevalence of NS use, the current study revealed that NS use was more prevalent among China's college students suffering from inflammatory skin diseases. Although there is at least moderate-quality evidence to support the effectiveness of NS for AD patients, the lack of long-term observation makes it challenging to determine whether NS is beneficial in real-world settings. Further research is required to elaborate on the reason or motivation for NS use. Given the fact that individuals with inflammatory skin diseases are more likely to use NS, health practitioners should be prepared to pay attention to the potential benefits and risks of various NS products in the context of inflammatory skin diseases.

Moreover, the public did not have enough understanding and the appropriate attitude about NS because it has only just become known over the past decade; therefore, government regulators and the scientific community should strengthen the supervision of NS industry and NS product marketing, widely publicize NS related information through various publicity channels to avoid false and exaggerated publicity, and then help people gain better understandings of NS.

## Data Availability Statement

The original contributions presented in the study are included in the article/Supplementary Material, further inquiries can be directed to the corresponding authors.

## Ethics Statement

This study was conducted according to the guidelines laid down in the Declaration of Helsinki. All procedures involving study participants were approved by the institutional research Ethics Board of Xiangya Hospital, Central South University (Changsha, China). Written informed consent was obtained from all participants before the investigation.

## Author Contributions

YY analyzed the data and drafted the manuscript. LJ, MS, and XC designed the study. JS, JL, JT, XK, BW, SS, and XW acted as study site coordinators. MS, JS, and XC obtained the funding. All authors participated in the field survey and data collection, critically revised the manuscript, and gave final approval to the version submitted for publication.

## Conflict of Interest

The authors declare that the research was conducted in the absence of any commercial or financial relationships that could be construed as a potential conflict of interest.
