# Supplementary material for: Behavior of Nutritional Supplements Use in Association With Inflammatory Skin Diseases in Chinese College Students
Source: Front Nutr. 2021 Mar 17;8:615462. doi: 10.3389/fnut.2021.615462 (PMC8010123; doi:10.3389/fnut.2021.615462)
Supplement: Supplementary file 1 [file Table_1.DOCX]

**Supplementary table 1. General demographic characteristics and types of NS taken by users**

| **Characteristics** | **Vitamin B** | | **Vitamin C** | | **Vitamin E** | | **Mineral** | | **Protein** | | **Herb** | |
| --- | --- | --- | --- | --- | --- | --- | --- | --- | --- | --- | --- | --- |
|  | **n(%)/Mean±SD** | **p Value** | **n(%)** | **p Value** | **n(%)** | **p Value** | **n(%)** | **p Value** | **n(%)** | **p Value** | **n(%)** | **p Value** |
| **Geographic region*** |  |  |  |  |  |  |  |  |  |  |  |  |
| North | 178(4.9) | ＜0.001 | 461(12.8) | ＜0.001 | 111(3.1) | ＜0.001 | 112(3.1) | 0.383 | 96(2.7) | ＜0.001 | 24(0.7) | 0.064 |
| Northeast | 62(9.8) |  | 120(19.0) |  | 28(4.4) |  | 30(4.8) |  | 23(3.6) |  | 11(1.7) |  |
| East | 224(5.0) |  | 631(14.0) |  | 158(3.5) |  | 143(3.2) |  | 123(2.7) |  | 31(0.7) |  |
| Central | 229(5.4) |  | 492(11.6) |  | 127(3.0) |  | 149(3.5) |  | 72(1.7) |  | 29(0.7) |  |
| South | 79(5.8) |  | 185(10.3) |  | 39(2.2) |  | 63(3.5) |  | 33(1.8) |  | 12(0.7) |  |
| Southwest | 194(10.8) |  | 556(13.9) |  | 97(2.4) |  | 145(3.6) |  | 148(3.7) |  | 29(0.7) |  |
| Northwest | 75(1.9) |  | 143(10.6) |  | 46(3.4) |  | 50(3.4) |  | 34(2.5) |  | 15(1.1) |  |
| **Age** | 18.3±0.6 |  | 18.3±0.7 |  | 18.2±0.7 |  | 18.2±0.6 |  | 18.4±0.8 |  | 18.3±0.9 |  |
| **BMI,( kg/m2)** |  |  |  |  |  |  |  |  |  |  |  |  |
| Underweight (<18.5) | 215(5.4) | 0.349 | 543(13.6） | 0.319 | 104(2.6) | 0.032 | 136(3.4) | 0.567 | 105(2.6) | 0.044 | 33(0.8) | 0.722 |
| Normal (18.5-23.9) | 605(5.0) |  | 1538(12.6) |  | 360(3.0) |  | 409(3.4) |  | 339(2.8) |  | 88(0.7) |  |
| Overweight (24.0-27.9) | 150(5.7) |  | 347(13.1) |  | 99(3.7) |  | 103(3.9) |  | 59(2.2) |  | 23(0.9) |  |
| Obese (≥28.0) | 58(5.7) |  | 124(12.1) |  | 38(3.7) |  | 33(4.8) |  | 16(1.6) |  | 6(0.6) |  |
| **Sex** |  |  |  |  |  |  |  |  |  |  |  |  |
| Male | 525(5.1) | 0.685 | 1121(10.9) | ＜0.001 | 281(2.7) | 0.02 | 376(3.7) | 0.09 | 270(2.6) | 0.98 | 63(0.6) | 0.021 |
| Female | 516(5.2) |  | 1467(14.9) |  | 325(3.3) |  | 316(3.2) |  | 259(2.6) |  | 88(0.9) |  |
| **Ethnicity** |  |  |  |  |  |  |  |  |  |  |  |  |
| Han | 892(5.5) | ＜0.001 | 2116(13.0) | 0.113 | 518(3.2) | 0.002 | 570(3.5) | 0.249 | 392(2.4) | ＜0.001 | 124(0.8) | 0.71 |
| Other | 149(3.8) |  | 472(12/1) |  | 88(2.3) |  | 122(3.1) |  | 137(3.5) |  | 27(0.7) |  |
| **Annual household income, (yuan)** |  |  |  |  |  |  |  |  |  |  |  |  |
| <10000 | 59(2.7) | ＜0.001 | 167(7.7) | ＜0.001 | 40(1.8) | ＜0.001 | 54(2.5) | ＜0.001 | 59(2.7) | ＜0.001 | 12(0.6) | ＜0.001 |
| 10000-29999 | 154(3.5） |  | 407(9.3) |  | 89(2.0) |  | 93(2.1) |  | 84(1.9) |  | 17(0.4) |  |
| 30000-49999 | 160(4.6) |  | 391(11.3) |  | 98(2.8) |  | 108(3.1) |  | 86(2.5) |  | 19(0.5) |  |
| 50000-99999 | 217(4.9) |  | 587(13.3) |  | 125(2.8) |  | 178(4.0) |  | 95(2.1) |  | 28(0.6) |  |
| 100000-199999 | 283(7.0) |  | 702(17.3) |  | 160(3.9) |  | 180(4.4) |  | 128(3.1） |  | 44(1.1) |  |
| ≥200000 | 168(10.3) |  | 334(20.4) |  | 95(5.7) |  | 79(4.8) |  | 77(4.7) |  | 31(1.9) |  |
| No | 831(5.2) |  | 2032(12.8) |  | 469(3.0) |  | 553(3.5) |  | 410(2.6) |  | 116(0.7) |  |
| Yes | 210(4..9) |  | 556(13.0) |  | 137(3.2) |  | 139(3.2) |  | 119(2.8) |  | 35(0.8) |  |
| **Frequency of exercise, (h/wk)** |  |  |  |  |  |  |  |  |  |  |  |  |
| 0-2 | 404(4.8) | 0.081 | 1036(12.2) | 0.042 | 239(2.8) | 0.292 | 262(3.1) | 0.029 | 193(2.3) | 0.002 | 64(0.8) | 0.211 |
| 2-7 | 229(5.4) |  | 548(12.9) |  | 141(3.30 |  | 144(3.4) |  | 103(2.4) |  | 24(0.6) |  |
| ≥7 | 408(5.5) |  | 1004(13.6) |  | 226(3.1) |  | 286(3.9) |  | 233(3.1) |  | 63(0.9) |  |
| **Sedentary activities, (h/d)** |  |  |  |  |  |  |  |  |  |  |  |  |
| Hardly | 88(5.5) | 0.15 | 193(12.1) | 0.091 | 43(2.7) | 0.411 | 44(2.8) | 0.078 | 41(2.6) | 0.655 | 13(0.8) | 0.931 |
| ＜7 | 441(4.8) |  | 1132(12.4) |  | 264(2.9) |  | 298(3.3) |  | 230(2.5) |  | 69(0.8) |  |
| ≥7 | 512(4.80 |  | 1263(13.4) |  | 299(3.2) |  | 350(3.7) |  | 258(2.7) |  | 69(0.7) |  |
| **Passive smoke exposure** |  |  |  |  |  |  |  |  |  |  |  |  |
| Hardly | 764(4.8) | ＜0.001 | 1940(12.2) | ＜0.001 | 428(2.8) | ＜0.001 | 503(3.2) | ＜0.001 | 395(2.5） | 0.015 | 110(0.7) | 0.082 |
| frequently | 277(6.5) |  | 648(15.3) |  | 168(4.0) |  | 189(4.5) |  | 134(3.2) |  | 41(1.0) |  |
| **Alcohol** |  |  |  |  |  |  |  |  |  |  |  |  |
| Hardly | 952(4.9) | ＜0.001 | 2424(12.0) | ＜0.001 | 566(2.9) | 0.01 | 643(3.3) | 0.001 | 486(2.5) | ＜0.001 | 143(0.7) | 0.623 |
| frequently | 89(9.9) |  | 164(18.2) |  | 40(4.4) |  | 31(7.1) |  | 43(4.8) |  | 8(0.9) |  |
| **milk** |  |  |  |  |  |  |  |  |  |  |  |  |
| 0 | 249(4.0) | ＜0.001 | 615(9.8) | ＜0.001 | 136(2.2) | ＜0.001 | 139(2.2) | ＜0.001 | 108(1.7) | ＜0.001 | 40(0.6） | 0.057 |
| 1 | 295(4.7） |  | 760(12.1) |  | 158(2.5) |  | 191(3.0) |  | 159(2.5) |  | 40(0.6) |  |
| 2 | 497(6.6) |  | 1213(16.0) |  | 312(4.1) |  | 362(4.8) |  | 262(3.5) |  | 71(0.9) |  |
| **yogt** |  |  |  |  |  |  |  |  |  |  |  |  |
| 0 | 213(3.8) | ＜0.001 | 479(8.7) | ＜0.001 | 107(1.9) | ＜0.001 | 139(2.5) | ＜0.001 | 111（2.0) | ＜0.001 | 34(0.6) | ＜0.001 |
| 1 | 415(4.7) |  | 1104(12.5) |  | 252(2.8) |  | 281(3.2) |  | 220(2.5) |  | 51(0.6) |  |
| 2 | 413(7.2) |  | 1005(17.5) |  | 247(4.3) |  | 272(4.7) |  | 198(3.5) |  | 66(1.2) |  |
